# Activity Time Budget during Foraging Trips of Emperor Penguins

**DOI:** 10.1371/journal.pone.0050357

**Published:** 2012-11-21

**Authors:** Shinichi Watanabe, Katsufumi Sato, Paul J. Ponganis

**Affiliations:** 1 Faculty of Life Science and Biotechnology, Fukuyama University, Fukuyama, Hiroshima, Japan; 2 International Coastal Research Center, Atmosphere and Ocean Research Institute, The University of Tokyo, Kashiwa, Chiba, Japan; 3 Center for Marine Biotechnology and Biomedicine, Scripps Institution of Oceanography, University of California San Diego, La Jolla, California, United States of America; Phillip Island Nature Parks, Australia

## Abstract

We developed an automated method using depth and one axis of body acceleration data recorded by animal-borne data loggers to identify activities of penguins over long-term deployments. Using this technique, we evaluated the activity time budget of emperor penguins (n = 10) both in water and on sea ice during foraging trips in chick-rearing season. During the foraging trips, emperor penguins alternated dive bouts (4.8±4.5 h) and rest periods on sea ice (2.5±2.3 h). After recorder deployment and release near the colony, the birds spent 17.9±8.4% of their time traveling until they reached the ice edge. Once at the ice edge, they stayed there more than 4 hours before the first dive. After the first dive, the mean proportions of time spent on the ice and in water were 30.8±7.4% and 69.2±7.4%, respectively. When in the water, they spent 67.9±3.1% of time making dives deeper than 5 m. Dive activity had no typical diurnal pattern for individual birds. While in the water between dives, the birds had short resting periods (1.2±1.7 min) and periods of swimming at depths shallower than 5 m (0.25±0.38 min). When the birds were on the ice, they primarily used time for resting (90.3±4.1% of time) and spent only 9.7±4.1% of time traveling. Thus, it appears that, during foraging trips at sea, emperor penguins traveled during dives >5 m depth, and that sea ice was primarily used for resting. Sea ice probably provides refuge from natural predators such as leopard seals. We also suggest that 24 hours of sunlight and the cycling of dive bouts with short rest periods on sea ice allow emperor penguins to dive continuously throughout the day during foraging trips to sea.

## Introduction

Penguins are widely distributed around the Southern Ocean, and they are important consumers in the marine ecosystem [1]. Some species are adapted to live in extreme environments, but they can be highly sensitive to climate change, which disrupts penguin life history strategies [2]. In particular, climate warming in the Antarctic region causes retreat of glaciers, collapse of large ice shelves, and consequently reduction of sea ice [3]–[6], which affect breeding population sizes and distribution ranges of penguins [2], [7], [8].Specific responses of penguins to climate changes varied among species, due to variability of adaptation of species to the environmental conditions. Current hypotheses suggest that sea ice reduction has produced habitat conditions more suitable for ice-intolerant species, which have increased in numbers, whereas ice-dependent species, closely linked to sea ice, have declined [8]. For example, the ice-intolerant species such as Gentoo penguins (*Pygoscelis papua*) have increased in number and expanded their range southward, while the ice-dependent species such as Adélie penguins (*Pygoscelis adeliae*) has decreased at almost all locations on the Antarctic Peninsula and contracted their distributional range poleward [2], [7], [9].

The emperor penguin (*Aptenodytes forsteri*) is considered an ice-dependent species, because it uses fast ice as breeding ground. For specific responses of emperor penguins to sea ice condition, the population dynamics and the breeding success have been studied [10], [11]. Although sea-ice condition affects the penguin populations, the relationship between the foraging behavior of penguins and sea-ice condition is not clear, due to difficulties of tracking behavior at sea and on sea ice.

Herein, we used animal-borne data loggers to monitor both activities in water and on sea ice of free-ranging emperor penguins during foraging trips. Yoda et al. [12] presented a method to identify behavioral activities of Adélie penguins using depth and bi-axial acceleration recorded by data loggers. The authors discriminated each behavior by visual observation of acceleration data. Recently developed loggers used in this study permit recording for a much longer period, up to ca. 15 days. To deal with such a large amount of data, a more convenient and effective classification method on acceleration data was needed. Sakamoto et al. [13] developed new software, Ethographer, to analyze and classify body acceleration data into several categories automatically. In the present study, we used a procedure based on Ethographer to automatically identify the penguin’s activity using depth and one axis of body acceleration data. Then, we evaluated the activity time budget and diurnal pattern of emperor penguins during foraging trips to sea. In addition, we investigated relationships between activities in water and those on the ice in order to assess the role of sea ice (travel vs. rest) during emperor penguins’ foraging trips.

**Table 1:** Summary of logger data recorded during foraging trips of chick-rearing emperor penguins at Cape Washington in the Ross Sea, Antarctica.

Bird ID	Logger type	Delay time(h)	Initial bodymass(kg)	Massgain(kg)	Trip duration(days)	No. of dives(>20 m)	Max. dive depth(m)	Data length (h)	
								from the colonyto ice edge	during foraging
CW01	W1000-PD2GT	8	29.0	No data	7.9	330	358	41.7	52.1
CW02	W1000-PD2GT	8	23.0	2.5	10.0	464	418	16.3	79.9
CW03	W1000-PD2GT	8	21.5	4.0	17.8	434	423	39.8	59.4
CW04	W1000-PD2GT	48	23.5	2.0	13.5	472	514	No data	93.7
CW07	W1000-PD2GT	96	24.0	2.5	16.1	440	476	No data	91.8
CW08	W1000-PD2GT	96	27.5	3.0	13.7	434	502	No data	94.5
CW09	W1000-PD2GT	96	24.0	2.5	15.0	427	500	No data	95.0
CW10	W1000L-PD2GT	4	25.5	5.0	19.7	1939	459	10.4	332.4
CW11	W1000L-PD2GT	4	22.0	0.5	16.5	2083	400	5.6	354.6
CW13	W1000-3MPD3GT	4	26.0	4.0	11.5	1283	509	11.9	254.0

**Figure 1:**
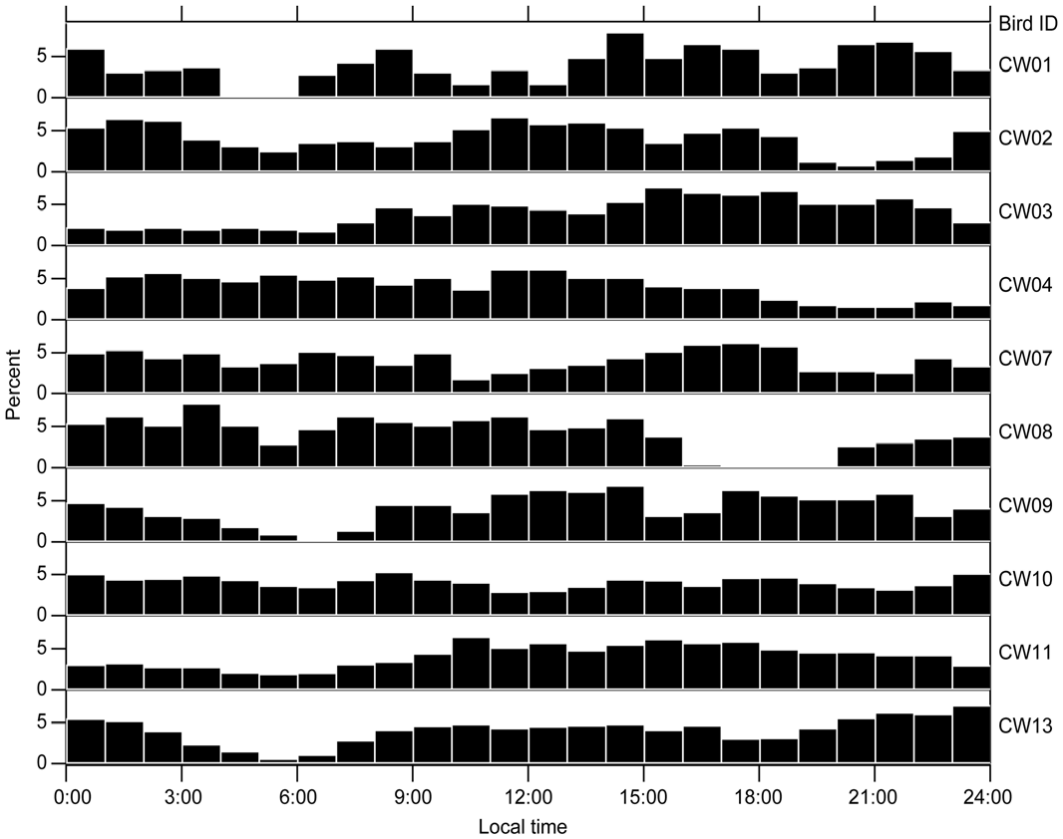
Time distribution in 24 h cycles of dives (≥20 m) for emperor penguins at Cape Washington in the Ross Sea, Antarctica.

## Results

Of 14 birds equipped with the loggers, one bird was not relocated after release and two birds did not depart for foraging trip. The remaining 11 birds were recaptured and instruments were retrieved, when they returned from their foraging trips to the colony. One of the 11 instruments did not work because of mechanical malfunction. Therefore, ten birds delivered reliable data of the foraging trips in this study. The foraging trip duration was 14.2±3.6 days (mean±*SD*). The birds had a mean mass of 24.6±2.4 kg, all of which were increased by 2.9±1.3 kg at the retrieval (Table 1). We obtained activity data for all ten emperor penguins during their foraging trips. For six birds, activities performed on their way from the colony to the ice edge were also recorded by the loggers (Table 1). All recordings ended during the foraging trips due to the limits of data storage capacity for the recorders.

The maximum dive depth of each bird ranged from 358 to 514 m (Table 1). Dives (>20 m) recorded in each bird were analyzed for time distribution over the 24 h day. The birds dived at any time through the 24 h cycle (Fig. 1). Typical diurnal patterns were not found among the birds.

**Figure 2:**
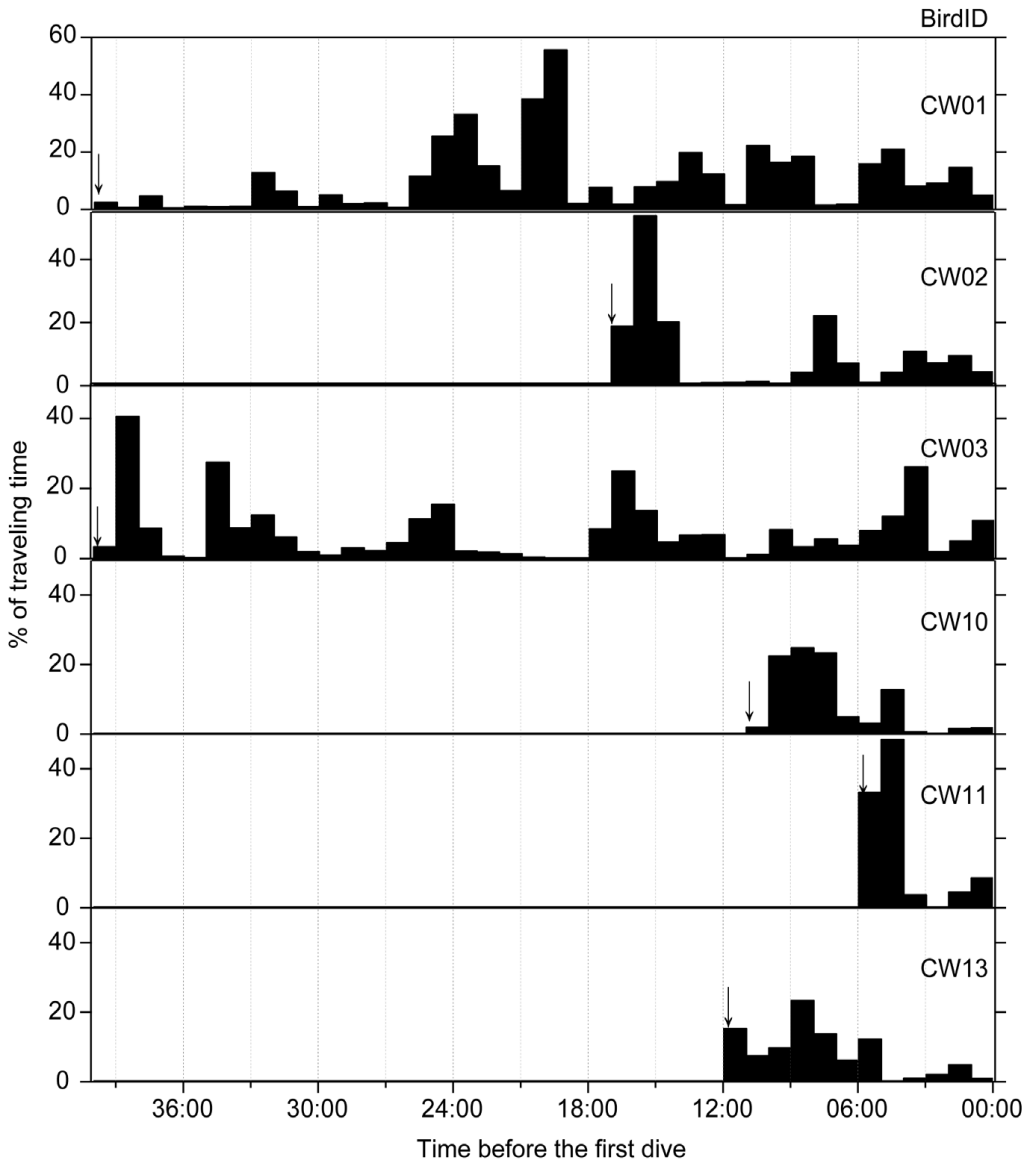
Percentages of traveling time in each hour for emperor penguins on the way from the colony to the ice edge at Cape Washington in the Ross Sea, Antarctica. Each arrow shows when recording was started.

For six birds of which activities on their way from the colony to the ice edge were recorded for 126 h in total (Table 1); the proportion of time spent in each activity is shown in Table 2. During the periods between the colony and the ice edge, traveling time, including walking and tobogganing, constituted 17.9±8.4% of the time. Walking predominated (93.8±2.4% of traveling time on the ice) over tobogganing. The percentages of traveling time before the first dive are shown in Fig. 2. Proportions of time spent for traveling were variable from the colony to ice edges. Periods with high proportions (>20%) of traveling time per hour were found in all birds. Such traveling periods were observed from 4 to 39 hours before the first dive; thus, they had stayed at the ice edge more than 4 hours before the first dive.

**Table 2:** Percentage of time spent in each activity between departure from the colony and arrival at the ice edge for emperor penguins at Cape Washington in the Ross Sea, Antarctica.

Bird ID	% of each activity between colony and ice edge			
	Stand	Lie	Walk	Toboggan
CW01	89.4	0.11	10.1	0.38
CW02	53.9	35.6	9.6	0.93
CW03	81.3	10.7	7.5	0.48
CW10	59.4	17.4	22.5	0.81
CW11	44.9	26.7	25.6	2.8
CW13	60.1	13.1	25.3	1.5
Mean ± S.D.	64.8±17	17.3±12.5	16.7±8.5	1.2±0.9

**Figure 3:**
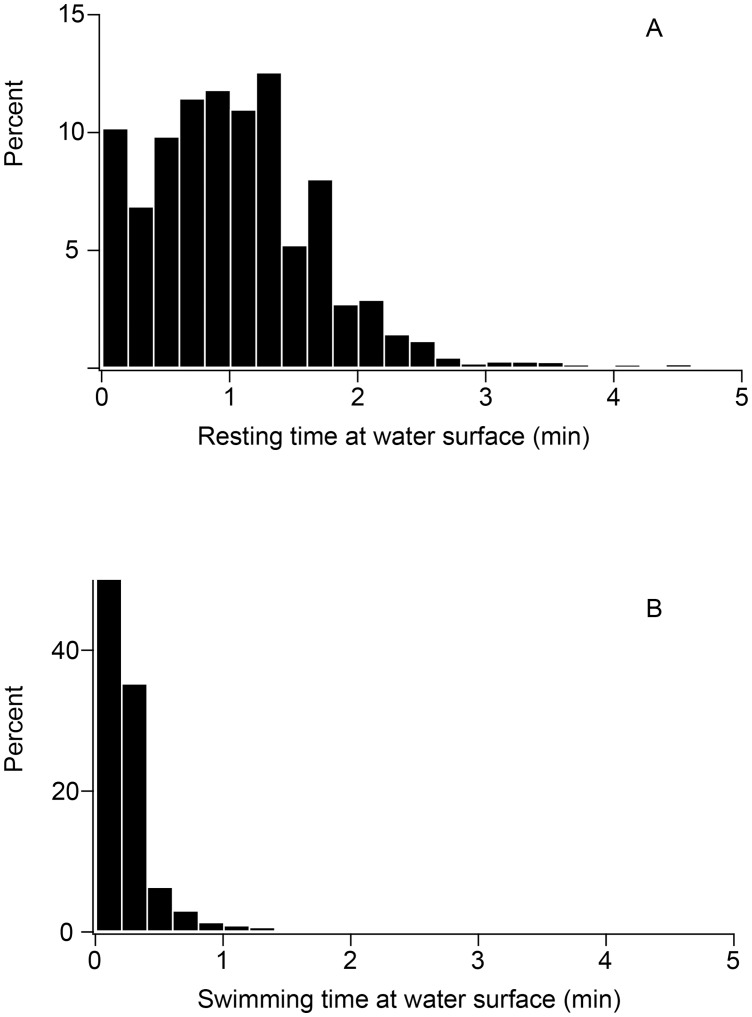
Frequency of the durations of rest periods (A) and swimming (B) at water surface (<5 m) between consecutive dives for emperor penguins.

After the first dive, the proportion of time spent on the ice and in water constituted 30.8% and 69.2%, respectively. The distribution of time spent in each activity during foraging trips is shown in Table 3. When birds were on the ice, they were inactive for the majority of time (90.3±4.1%): 62.9±10.1% of the inactive time was spent standing, and 37.1±10.1%, lying down. Traveling on the ice constituted only 9.7±4.1% of the time on the ice. Walking predominated (90.2±4.6% of traveling time on the ice) over tobogganing (9.8±4.6% of the traveling time on the ice). When the birds were in water, they spent 67.9±3.1% of time diving, with 45.6±3.9% of the dive time in the bottom phase. Surface swimming occupied 4.7±2.0% of the time in water, while resting at the surface was 27.3±3.6%. When the birds were at the water surface, they spent 1.2±1.7 min resting and 0.25±0.38 min swimming before the next dive (Fig. 3); 92.8% and 99.6% of surface rest and swim periods were less than 2 min, respectively.

**Table 3:** Percentage of time spent in each activity during foraging trips for emperor penguins at Cape Washington in the Ross Sea, Antarctica.

Bird ID	% of each activity during foraging trip										
	On ice					In water					
	Stand	Lie	Walk	Toboggan	Total	Rest	Swim	Descent	Bottom	Ascent	Total
CW01	16.2	4.0	2.9	0.19	23.2	20.2	4.4	15.0	25.2	12.0	76.8
CW02	14.6	9.0	1.8	0.02	25.4	23.2	2.3	15.4	20.7	13.0	74.6
CW03	7.7	8.5	3.4	0.19	19.8	19.1	1.7	17.4	26.2	15.7	80.2
CW04	17.9	13.7	1.9	0.29	33.8	19.0	3.6	13.5	18.3	11.8	66.2
CW07	19.7	5.3	1.5	0.24	26.7	22.1	3.5	15.0	19.0	13.7	73.3
CW08	24.2	13.3	5.0	0.52	43.0	16.4	1.6	9.5	20.0	9.4	57.0
CW09	17.1	11.3	2.0	0.32	30.7	22.5	2.6	11.9	19.5	12.7	69.3
CW10	17.2	12.9	1.6	0.31	32.0	17.6	2.5	12.8	23.1	12.0	68.0
CW11	21.6	16.6	2.7	0.33	41.2	12.2	4.8	10.4	21.2	10.1	58.8
CW13	18.4	9.7	3.2	0.31	31.7	17.3	5.1	13.4	20.7	11.9	68.3
Mean ± S.D.	17.5±4.4	10.4±3.9	2.6±1.1	0.27±0.13	30.8±7.4	19±3.3	3.2±1.3	13.4±2.4	21.4±2.6	12.2±1.8	69.2±7.4

Results based on dive bouts were summarized in Table 4. Durations of dive bout varied widely. 42.9% and 67.2% of dive bouts were less than 2 h and 5 h, respectively. After the dive bout, the birds spent 2.5±2.3 h on the ice before the next dive bout. 68.7% and 76.8% of ice rest periods were less than 2 h and 3 h, respectively. Traveling time and estimated distance traveled on the ice between dive bouts were ranging from 4 sec to 2.9 h) and in 7–5430 m), respectively.

**Table 4:** Number (No.) and mean ± SD duration of each activity during dive bouts and during time on ice during foraging trips of emperor penguins from Cape Washington in the Ross Sea, Antarctica.

Bird ID	During dive bouts			During time on ice		
	No. of dive bouts	No of dive bouts per day	Duration of divebout (h)	Time spent on ice (h)	Traveling time(min)	Estimated Traveling distance (m)
CW01	5	2.4	7.4±4.7	2.4±2.1	19.3±4.3	592±125
CW02	7	2.2	8.2±6.2	2.9±1.5	12.2±4.2	368±128
CW03	5	2.1	9.2±4.0	2.4±1.8	25.7±12.3	786±376
CW04	7	2.0	7.9±5.0	4.4±3.1	15.8±8.2	501±252
CW07	7	2.0	8.2±5.7	3.5±4.2	13.6±6.4	429±196
CW08	13	3.4	4.0±3.2	3.1±2.8	24±16.8	747±514
CW09	6	1.6	10.0±5.7	4.6±2.5	19.6±8.3	617±261
CW10	43	3.1	5.2±5.1	2.5±2.1	9.0±6.9	286±213
CW11	64	4.4	3.2±3.4	2.3±2.0	9.9±22.6	311±699
CW13	41	4.2	3.8±3.5	2.0±1.9	13.1±11.3	407±343
Mean ± S.D.		2.7±1.0	4.8±4.5	2.5±2.3	12.7±15.7	395±484

Results of LMMs to investigate relationships between activities on the ice and those during previous dive bouts are shown in Table 5. For the models with resting time on the ice as dependent variable, AICc of the models with each of duration of dive bouts, accumulated time of diving, and accumulated time of bottom time duration as a fixed effect were much smaller than that of the model including only the random effect (Bird ID); the AICc differences were more than 5.0. The most parsimonious model with the lowest AICc has five times greater *wi* than those of other models (*Resting time on ice* = 0.1271 × *duration of previous dive bout* +0.17280), which showed that resting time on the ice increased with duration of dive bouts. On the other hand, for the models with traveling time on the ice as dependent variable, difference of AICc between models with any fixed effect and random effect only was lower than 2.0. In addition, the *wi* of each model was lower than 0.4. Thus, resting time on the ice was best explained by the time duration of the previous dive bout. On the other hand, traveling time on the ice between dive bouts was not clearly related to one single factor: the duration of the previous dive bout, the cumulative diving time, and the cumulative bottom time each gave similar model fits.

**Table 5:** Results of fitting of linear mixed models (LMM) for resting and traveling time on sea ice between dive bouts during foraging trips of emperor penguins.

Fixed effect	Dependent variable					
	Resting time on ice			Traveling time on ice		
	AICc	delta AICc	Akaike weight(*wi*)	AICc	delta AICc	Akaike weight(*wi*)
Duration of dive bout	861.0	0	0.799	28.3	0	0.319
Accumulated time of diving	864.3	3.3	0.152	28.6	0.29	0.276
Accumulated time of bottom time duration	866.7	5.7	0.047	28.6	0.28	0.278
None (only random effect)	872.4	11.4	0.003	30.2	1.84	0.127

## Discussion

This is the first report for the activity time budget both in water and on sea ice of breeding emperor penguins on foraging trips during chick-rearing period. Our results showed that emperor penguins made repeated dive bouts and rest periods on sea ice over a prolonged period. Although it is known that emperor penguins perform repeated dive bouts during foraging trips at sea [14], the detailed activity of each individual during a trip, especially when they are on the ice, has not been reported previously.

Our results show that the penguins primarily rested on sea ice. Continuous travel over long distances did not occur on sea ice. We also found that after leaving the colony and arriving at the ice edge prior to the start of a foraging trip, birds spent 3 to 38 hours waiting before making the first dive. We suspect that rest on the sea ice and prolonged waits at the ice edge are secondary to the presence of predators. For Antarctic penguins, predation risks by leopard seals (*Hydrurga leptonyx*) are high at sea ice edges [15], and seals tend to aggregate at the ice edge where departing penguins congregate [15], [16]. It is known that Antarctic penguins form a temporal flock and synchronously dive from ice edge [e.g.,15,17]. The risk of predation might be reduced when penguins start dives synchronously, through dilution effects or increased vigilance against seals [17]. Thus, it is more likely that such a stay at ice edge is the result of waiting for other individuals to reach the ice edge and form a flock for diving. This is supported by personal observation by KS and PJP. (see Movies S1 and S2).

During the foraging trip itself, the penguins spent 31% of time on sea ice. How do they rely on the sea ice during the foraging trip? From our data, the birds primarily rested while they were on sea ice. Traveling time on the ice was only 9.7% of the total time on the ice. In addition, mean traveling distance between dive bouts was only 395 m. Shiomi et al. [18] used the 3-D loggers to estimate diving paths of emperor penguins and reported that emperor penguins traveled horizontal distances in excess of 400 m on average during a dive, with a maximum of 1211 m. Thus, it is most likely that the birds move significant distances during dives at sea. This conclusion is further supported by the fact that it is more cost-efficient to move while diving than surface swimming or walking [19].

While there was no significant relationship between travelling time on sea ice and activities during the previous dive bout, rest time on the ice did correlate with the duration of the previous dive bout. It is thus likely that birds exit onto sea ice after they are fatigued by diving. Although birds rested at the sea surface, rest periods in the water were much shorter, mostly less than 2 min, than those on sea ice. It is more likely that these short breaks at the sea surface are not secondary to fatigue but are due to increased respirations for gas exchange. Therefore, it is more likely that emperor penguins use sea ice for resting than for traveling during foraging trips. Rest on the sea ice and away from the ice edge allows for avoidance of predators. In an exception to these observations, one bird spent 38% of time on ice for traveling and covered over 5 km on the ice between dive bouts. It may be that ice conditions prevented this bird’s return to the water, and that it therefore traveled over sea ice.

For foraging patterns of many species of penguins, typical diurnal patterns are observed as they leave the colony after sunrise and spend time foraging in the sea during the daytime and return to the colony before sunset [14]. Foraging trips of penguins living in lower latitude regions are observed most frequently around noon when the light intensity is high [e.g. 20–22]. Presumably, penguins dive and forage at times of day with sufficient light as they visually search prey [14]. In addition, recent studies show that Antarctic penguins are capable of successful prey capture in the dark but that they may avoid foraging at night due mainly to fear of predation [23]. By contrast, at our study site, the high latitude region during austral summer, the sun always appears throughout the day. From our data, no typical diurnal patterns were observed among individuals; the birds dived at any time through the 24 h cycle. In another study in the same region as our investigation, the birds dived all the day and clear diurnal patterns were not found, although deep dives more than 400 m were limited to periods with relatively greater light [24]. It may be that there are some diurnal patterns in foraging behavior of emperor penguins, but it is not obvious in comparison to those of other species of penguins living in the lower latitude regions.

There are a few reports studying activity time budgets of penguins both in water and out of water. Yoda et al. [12] compared activity time budgets of Adélie penguins between two different sites where sea ice is abundant and scarce. The results show that the birds spent 35% of the foraging trip in water of which 91% of time was spend diving during abundant sea ice conditions, while they spent 88% of time in water of which 55% of time was spend diving during scarce sea ice condition [12]. In our results, the emperor penguins spent much more time diving compared to Adélie penguins with shorter foraging trips; emperor penguins spent 69% of the time in water of which 66% of time was spent diving. We suggest that 24 hours of sunlight and the cycling of dive bouts with short rest periods on sea ice allow emperor penguins to dive continuously throughout the day during prolonged foraging trips to sea.

## Methods

### Field Experiments

The field study was conducted at the breeding colony at Cape Washington (74°39′S, 165°24′E) during the chick-rearing period from 26 October to 24 November 2005. Fourteen birds departing the colony were captured near the edge of the colony. Data loggers were attached to the central back feathers using waterproof Tesa™ tape (Beiersdorf AG, Hamburg, Germany) and stainless steel cable ties. Eleven birds were equipped with acceleration data loggers (W1000-PD2GT: 22 mm diameter, 122 mm in length, 73 g mass in air or W1000L-PD2GT: 27 mm diameter, 128 mm in length, 101 g mass in air, Little Leonardo, Tokyo, Japan) which recorded depth (1 Hz), temperature (1 Hz), speed (1 Hz), and two axes of acceleration (16 Hz, respectively). Three birds were equipped with 3-D loggers (W1000L-3MPD3GT: 26 mm diameter, 174 mm in length, 120 g mass in air, Little Leonardo Ltd, Tokyo, Japan) which recorded depth (1 Hz), temperature (1 Hz), three axes acceleration and three axes geomagnetism (8 Hz, respectively). The devices were set to start recording 4–96 h after deployment by the preset delay timer. According to the limitation of memory size, the logger cannot cover the whole foraging trip of each individual. The variation of recording start time (4–96 h) enabled us to record all phases of the foraging trips. VHF transmitters (Model MM130, ATS, Isanti, MN, USA) were attached to the lower back with cable ties. Body mass was measured to the nearest 100 g at deployment and recapture, using a balance (Pesola 50 kg). Mass of the devices deployed were less than 1% of the body mass. Methods of the field study and the deployments of loggers were described in detail in [25].

### Identification of Activity Types

**Figure 4:**
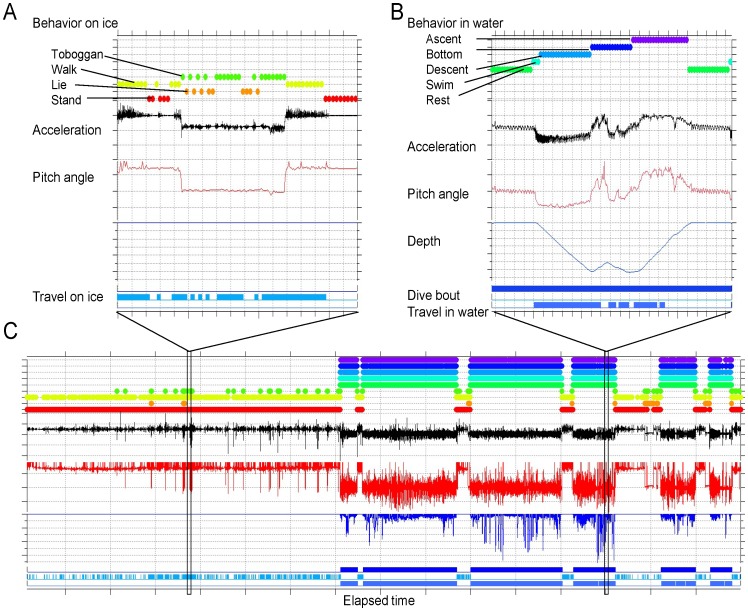
An example of classification results for activities on sea ice (A) and in water (B) during ca. 4 days recording (C) on an emperor penguin (CW01).

Penguins’ activities were categorized every 4 seconds using the depth and longitudinal axis of acceleration (Fig. 4). We put as few parameters as possible into the analysis, because we aimed to provide a simple procedure, which is potentially applicable to estimate activity in various species of aquatic animals.

Loggers measured both specific acceleration (such as walking, tobogganing, or swimming) and gravity-related acceleration [12]. Low-frequency components (<0.5 Hz) of the fluctuation in longitudinal acceleration, along the long axis of the body, were used to calculate the pitch angle of the animal [26].

**Figure 5:**
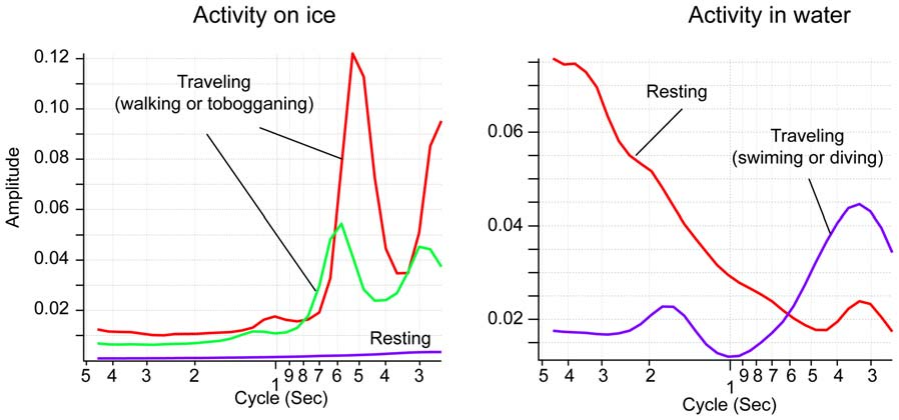
Examples of results of k-means cluster analysis on the spectral components of body acceleration recorded on an emperor penguin.

**Figure 6:**
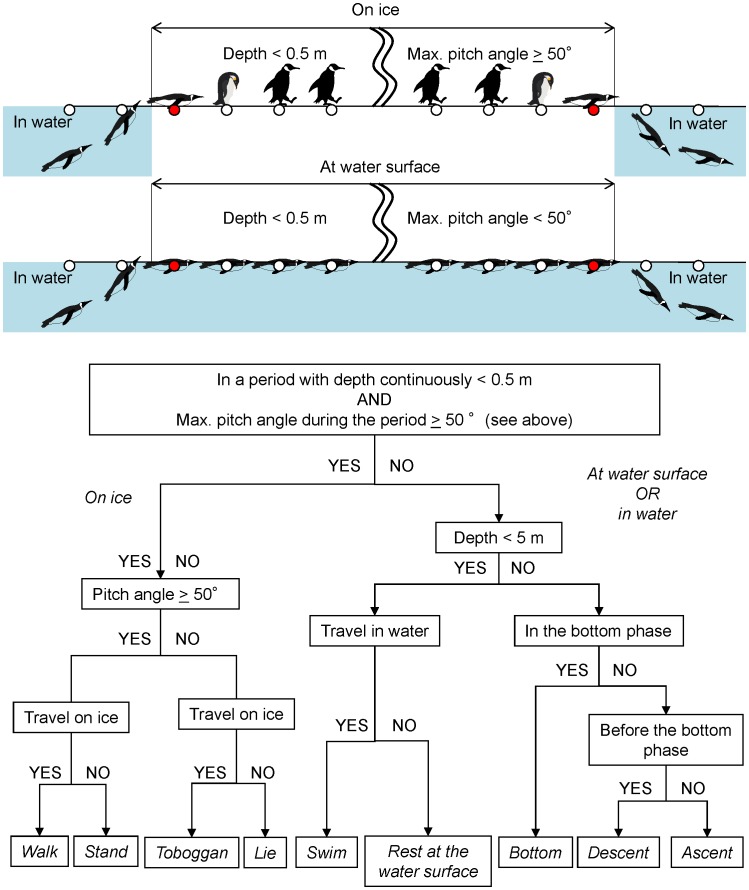
A flowsheet to categorize nine activity types of emperor penguins by time (lower) and a diagram of the first step in the procedures (upper), using depth data and two components of acceleration data along with the longitudinal axis of the body recorded by a logger. Pitch angle was estimated from the low-frequency component less than 0.5 Hz and traveling activity estimated from high-frequency component in the k-means cluster analysis.

Data retrieved from the loggers were analyzed using a customized macro program in IGOR PRO version 6 (WaveMetric, Inc., Lake Oswego, OR, USA). We also used Ethographer [13], a plug-in software in IGOR, to derive the high-frequency component resulting from specific activities of penguins. The acceleration data were converted into a spectrum every 4 seconds by continuous wavelet transformation. Then, each spectrum was categorized into one of behavior categories by unsupervised cluster analysis using k-means methods. The typical behaviors extracted were characterized by the periodicities of body acceleration [12]. Each categorized behavior was assumed to correspond to when the bird was traveling on ice (walking or tobogganing) and in water (swimming on the water surface or diving) (Fig. 5). Spectral analysis and k-means clustering were carried out on data on ice and in water, respectively. The behaviors classified by the procedures accorded well with those independently defined from depth and pitch angle [13], and nine activity types were identified from the decision tree analysis (Fig. 6).

**Figure 7:**
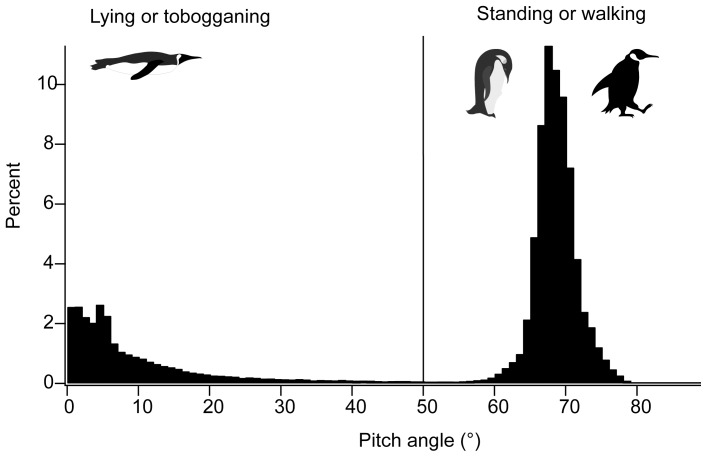
Histogram of pitch angles estimated on an emperor penguin on sea ice. When the bird was standing or walking, the pitch angle was greater than 50 degree.

In the procedures, it was first determined whether a bird was on ice or at the water surface (depth <0.5 m). Then, the maximum pitch angle during a period between the first and end points at which depths were less than the threshold (depth = 0.5 m) was calculated. A pitch angle of ≥50° was used as a criterion for walking or standing because the distribution of pitch angle was bimodal with higher values (≥50°) corresponding to walking and standing on ice (Fig. 7). If the maximum pitch angle during a period (depth <0.5 m) greater than 50°, i.e. standing or walking behavior was identified in that period, the bird was then classified as being on the ice.

**Figure 8:**
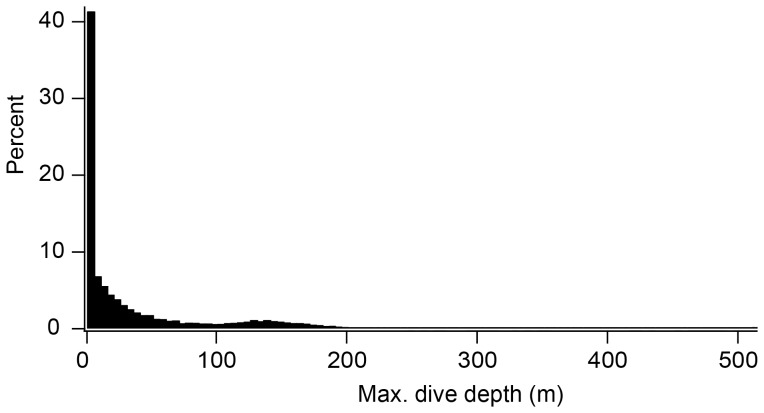
Histogram of maximum dive depth for all dives (maximum depth ≥1); 40% of all dives were <5 m in depth.

When a bird was on the ice, activities were categorized into four types according to the pitch angle and traveling activity derived by the k-means cluster analysis (Fig. 5, see above). If the pitch angle was greater than 50° and traveling activity was detected or not detected, the bird was categorized as walking or standing, respectively. On the other hand, if the pitch angle was lower than 50° and traveling activity was detected or not detected, the bird was categorized as tobogganing or lying at rest, respectively. When a bird was in water, activities were classified as at the surface (depth <0.5 m), submerged (or “surface”) swimming (maximum depth ≥0.5 m but <5 m), or diving (maximum depth ≥5 m). Submerged swimming near the surface constituted 40% of all dives (maximum depth ≥1 m) (Fig. 8). When a bird was in the shallow depth, activities were distinguished whether the bird was resting at water surface or swimming in the shallow depth according to traveling activity detected by k-means cluster.

When the bird was diving (the depth ≥5 m), activities were categorized into three types: descent, bottom, and ascent phases, according to the time series analysis of depth data. Bottom time, which was assumed to be the time spent in the foraging area, was defined as the duration from the first ascent to last descent in each dive [26]. The descent phase was time from the beginning of dive until the beginning of the bottom phase, and the ascent phase was time after the bottom phase until the end of dive.

### Statistical Analyses

Statistical analysis was performed based on dive bouts. If a bird exited the water and was on ice, the next dive was placed into a new dive bout. During the time between deployment/release near the colony and arrival at the ice edge, the proportion of time spent in each activity was calculated. When the bird was on the ice, traveling distance was estimated with accumulative time and mean walking speed (0.5 ms^−1^ [27]) and tobogganing (0.7 ms^−1^ [24]) of this species.

To investigate relationships between activities on the ice and the previous dive bout, we used a linear mixed model (LMM) fit by maximum likelihood with a Gaussian error distribution, identity link function, and bird ID as random effect for the intercept. When a bird stayed on the ice until the next dive bout, accumulative time for resting: standing and lying on the ice, and accumulative time for traveling: walking and tobogganing, were calculated and respectively compared with time duration, accumulated time of diving, and bottom time duration during the previous dive bout. Models were ranked based on their Akaike Information Criteria corrected for small sample sizes (AICc) and we calculated the Akaike weight (*wi*) for each model, which represents the relative likelihood of candidate models [28]. The most parsimonious model was considered to be the one with the lowest AICc value and the highest *wi* [28]. The LMM was performed with R 2.9 software (The R Foundation for Statistical Computing) and R package lme4 and function lmer [29].

## Supporting Information

Movie S1 **It shows a group of emperor penguins synchronously diving from ice edge.**
(MOV)

Movie S2 **It shows a leopard seal looking for emperor penguins at the edge of the water.**
(MOV)
